# Associations of body mass index, fasting insulin, and inflammation with mortality: a prospective cohort study

**DOI:** 10.1038/s41366-022-01211-2

**Published:** 2022-08-27

**Authors:** Natasha Wiebe, Paul Muntner, Marcello Tonelli

**Affiliations:** 1 Department of Medicine, University of Alberta, Edmonton, AB Canada; 2 School of Public Health, University of Alabama at Birmingham, Birmingham, AL USA; 3 Department of Medicine, University of Calgary, Calgary, AB Canada

**Keywords:** Epidemiology, Risk factors

## Abstract

**Background/objectives:**

Obesity is often considered to increase the risk for premature mortality. Higher fasting insulin and c-reactive protein are associated with higher body mass index (BMI) and all-cause mortality, so may confound the association between obesity and mortality. Our objective was to determine the independent associations between BMI, fasting insulin, c-reactive protein, and all-cause mortality in a general population sample.

**Methods:**

This prospective cohort study included non-institutionalized US adults (≥20 years) from the National Health and Nutrition Examination Surveys 1999–2000 to 2013–2014. The main exposures of interest were BMI, fasting insulin, c-reactive protein. Mortality data were obtained through linking participants to the National Death Index (ending December 31, 2015).

**Results:**

There were 12,563 participants with a median age of 45 years (range 20–85) and 47.9% were male. The median BMI was 27 kg/m^2^ (IQR 24–32), median fasting insulin was 54 pmol/L (IQR 35–87), and median c-reactive protein was 1.9 mg/L (IQR 0.8–4.4). In a Cox model adjusted for age, biological sex, cigarette smoking, and ten chronic conditions, higher BMI parameterized with quadratic and linear terms was not associated with mortality. When fasting insulin and the natural logarithm of c-reactive protein were included in the model, an inverse association between BMI and mortality was present (compared to the referent category of 5th percentile: 1st percentile, HR 1.10, 95% CI 1.06-1.13; 99th percentile, HR 0.48, 95% CI 0.34–0.69). In contrast, higher levels of fasting insulin and c-reactive protein were associated with an increased risk of mortality (for fasting insulin: 1st percentile, HR 0.98, 95% CI 0.97–0.99; 99th percentile, HR 1.83, 95% CI 1.48–2.26; for c-reactive protein, 1st percentile, HR 0.87, 95% CI 0.84–0.90; 99th percentile, HR 2.77, 95% CI 2.12–3.62).

**Conclusions:**

Higher fasting insulin and higher c-reactive protein confound the association between BMI and the risk of all-cause mortality. The increase in mortality that has been attributed to higher BMI is more likely due to hyperinsulinemia and inflammation rather than obesity.

## Introduction

Obesity is associated with an increased prevalence and incidence of many noncommunicable chronic diseases (NCDs) including end-stage kidney disease [1, 2], type II diabetes [3], cardiovascular disease [3, 4], atrial fibrillation [5], nonalcoholic steatohepatitis [6], and colorectal cancer [7–10]. However, among populations with these NCDs the risk of mortality is often lower for those with obesity [1–10]. It is also associated with a lower risk of mortality among elderly people and those who are critically ill [11–13]. Obesity is often uncritically accepted as a cause of disease and premature mortality, although this putative causal link does not meet the Bradford Hill criteria [14, 15]. In 2012, the American Medical Association’s Council on Science and Public Health agreed that there was “a lack of evidence of any true causal relationship between obesity and morbidity and/or mortality;” [16] notwithstanding, the American Medical Association resolved that obesity should be considered a disease state. Furthermore, major efforts such as the Global Burden of Disease study frequently claim that obesity is responsible for excess deaths without any further work establishing obesity as a causal agent. The authors of a Global Burden of Disease report stated that in 2017, high BMI caused 2.4 million deaths [17].

Hyperinsulinemia [18] and inflammation [19] have been postulated to be mediators between obesity and mortality. However, there have been few, if any, mediation analyses examining these factors in the pathway between BMI and mortality. Further, changes in insulin have been shown to precede changes in weight, but the converse has not been shown [20]. This suggests that obesity mediates an association between hyperinsulinemia and mortality rather than the other way around.

In this study, we evaluated the independent associations of BMI, fasting insulin, and c-reactive protein with all-cause mortality in a general population sample of US adults.

## Materials/subjects and methods

This secondary analysis of a prospective cohort study is reported according to the STROBE guidelines [21]. The institutional review boards at the Universities of Alberta (Pro00117530) and Calgary (pSite-22-0007 Pro00117530) approved this study. Written informed consent was obtained. The data are publicly available (https://wwwn.cdc.gov/nchs/nhanes/Default.aspx and https://www.cdc.gov/nchs/data-linkage/mortality-public.htm).

### Data sources

We used eight cycles of the publicly available National Health and Nutrition Examination Surveys (NHANES; 1999–2000 to 2013–2014) and linked them with the publicly available National Center for Health Statistics (NCHS) 2015 mortality file [22] to create an initial larger cohort (i.e., the BMI cohort). Only six NHANES cycles from 1999–2000 to 2009–2010 had data on c-reactive protein (CRP) and fasting insulin and we used these data to form the primary cohort. NHANES is a series of surveys conducted on non-institutionalized US adults identified through stratified four-stage probability random sampling. Through interviews and physical examinations, the NHANES collects information on demographics, physiological measurements, medical history, medication use, and laboratory tests.

### Population

The database was used to assemble two cohorts of adults (age ≥20 years) residing in the United States who had a measure of BMI and vital status ascertained as of December 31, 2015. This first cohort (the BMI cohort) included participants with complete data on biological sex, BMI, and vital status. The second cohort, a subset of the BMI cohort (the primary cohort), included participants with complete data on age, biological sex, BMI, fasting insulin, CRP, and vital status.

### Primary exposures

The exposures of interest were CRP, fasting insulin. and BMI. If a participant fasted for <8 or >24 h, their fasting insulin and glucose values were not used. CRP was assayed using latex-enhanced nephelometry. Its lower detection limit was 0.2 mg/L. The NHANES quality control and quality assurance protocols met the 1988 Clinical Laboratory Improvement Act requirements.

### Outcome

All-cause mortality was the study outcome. The NCHS used 13 variables to probabilistically ascertain vital status of survey participants: social security number, first name, middle initial, last name, father’s surname, month of birth, day of birth, year of birth, state of birth, state of residence, sex, race, and marital status.

### Covariates

Age, biological sex, cigarette smoking, and ten self-reported chronic conditions (angina, arthritis, diabetes, cancer, chronic heart failure, chronic liver disease, chronic lung disease, coronary artery disease, stroke, and thyroid problems) were included as covariates. Specifically, the participants were asked if a doctor or another health professional ever indicated that they had a particular condition. The ten chronic conditions were selected based on their availability in the datasets, and their associations with obesity. Fasting glucose, and glycated hemoglobin [HbA1c]) were considered in sensitivity analyses along with insulin resistance. Insulin resistance was measured using the Homeostatic Model Assessment [23] (HOMA-IR).

### Statistical analyses

We did the analyses with Stata MP 17.0 (www.stata.com) and reported baseline descriptive statistics as percentages, or medians and inter-quartile ranges, as appropriate. The NHANES uses a four-stage probability random sampling procedure (counties, city blocks, households, then individuals) and they oversample low-frequency demographic subgroups. In order to produce nationally representative results and to account for the sampling design (unequal probability of selection), we applied sampling weights. The variance was estimated using Taylor linearization.

In order to characterize the association between BMI and mortality, we used the larger BMI cohort. Time to mortality was regressed on BMI, parametrized with restricted cubic splines, and interacted with biological sex using Cox regression models. These unadjusted hazard ratios with 95% confidence intervals were plotted against BMI, and underlaid with the distributions of BMI by biological sex. Standard errors by biological sex were calculated.

Using the primary cohort, medians and inter-quartile ranges of fasting insulin and CRP are presented by BMI categories (<18.5, 18.5–<25, 25–<35, 35–<45, ≥45 kg/m^2^) and by biological sex. Differences by sex were tested with gamma or linear regression, as appropriate. Correlations between fasting insulin, CRP, and BMI were calculated. The distributions of BMI were plotted in pie charts for participants in the top 25th percentiles of fasting insulin and CRP, respectively.

Using the primary cohort, time to mortality was regressed on all three exposures of interest: BMI, fasting insulin, and CRP. A number of parametrizations of these three exposures were explored. The models with the best fit used linear and quadratic terms for BMI, a linear term for fasting insulin, and the natural logarithm of CRP. Model 1 adjusted for BMI, age, biological sex, and cigarette smoking (categorized as yes, no, and missing). Model 2 was further adjusted for fasting insulin and CRP. Model 3 further adjusted for the ten chronic conditions listed earlier.

We did four sensitivity analyses based on the fully adjusted model 3. First, we added glycated hemoglobin to model 3. Second, we added fasting glucose to model 3. Third, we replaced fasting insulin in model 3 with the HOMA-IR. Fourth, we replaced BMI in model 3 with percentage fat mass in further models.

To separate the effects of fasting insulin and CRP on the association of BMI with mortality, we used model 3 to evaluate every permutation of these three exposures, alone or in combination.

To explore whether the extent of inflammation might further modify the associations between fasting insulin, BMI, and mortality, we categorized participants as having CRP ≤ 10 mg/L vs >10 mg/L (none/low vs moderate/high grade of inflammation [24]). We then interacted the presence or absence of a moderate/high grade of inflammation with BMI and fasting insulin (model 4). The natural logarithm of CRP remained in this model as a main effect only.

Finally, we interacted with biological sex with the three exposures in the most adjusted third model (model 5).

All models, using the primary cohort, expressed hazard ratios with 95% confidence intervals for the 1st, 50th, 75th, 95th, and 99th percentiles of the three exposures as compared to a referent category of the 5th percentile. The 5th percentile was selected as the referent as a BMI of 20 kg/m^2^ corresponds with a so-called “healthy” BMI. We determined that the proportional hazard assumption was satisfied by examining plots of the log-negative-log of within-group survivorship probabilities versus log-time. The threshold two-sided *p* for statistical significance was set at 0.05.

## Results

### Characteristics of study participants

The application of inclusion criteria is shown in Supplementary Fig. S1. Of 43,494 adult participants, 40,677 had data on BMI and vital status (BMI cohort), and 12,563 had data on BMI, fasting insulin, CRP, ten chronic conditions, and vital status (primary cohort). The two cohorts had similar characteristics (Table 1). In both cohorts, women were more likely to have arthritis, thyroid problems, cancer, chronic lung disease and stroke than men. Men were more likely to have coronary artery disease, chronic liver disease, angina, and chronic heart failure. Men had higher levels of HOMA-IR, fasting glucose, and insulin and women had higher levels of CRP.

**Table 1:** Demographics and clinical characteristics by cohort.

Characteristic	BMI cohort			Primary cohort		
	All	Women	Men	All	Women	Men
*N*	40,677	21,115	19,562	12,563	6543	6020
Age, years	45 [33,59]	46 [33,60]	45 [32,57]	45 [33,58]	45 [33,59]	44 [32,56]
Smoking status	22.5	19.7	25.6	23.0	19.9	26.2
Physical and lab measures						
Body mass index, kg/m^2^	27 [24,32]	27 [23,33]	28 [25,31]	27 [24,32]	27 [23,32]	28 [24,31]
Fasting insulin, pmol/L	55 [36,88]	53 [35,84]	57 [37,92]	54 [35,87]	52 [34,82]	57 [37,92]
Fasting glucose, mmol/L	5.4 [5.1,5.9]	5.3 [4.9,5.8]	5.6 [5.2,6.0]	5.4 [5.0,5.8]	5.3 [4.9,5.7]	5.5 [5.2,6.0]
HOMA-IR	2.24 [1.39,3.82]	2.10 [1.32,3.56]	2.41 [1.48,4.09]	2.21 [1.38,3.75]	2.03 [1.29,3.46]	2.41 [1.48,4.04]
HbA1c, %	5.4 [5.1,5.7]	5.4 [5.1,5.7]	5.4 [5.1,5.7]	5.3 [5.1,5.6]	5.3 [5.1,5.6]	5.4 [5.1,5.6]
CRP, mg/L	1.9 [0.7,4.4]	2.4 [0.9,5.6]	1.5 [0.7,3.4]	1.9 [0.8,4.4]	2.4 [0.9,5.7]	1.5 [0.7,3.4]
Chronic condition	0 [0,1]	0 [0,1]	0 [0,1]	0 [0,1]	0 [0,1]	0 [0,1]
Angina	2.5	2.2	2.7	2.5	2.0	3.1
Arthritis	23.9	28.0	19.5	24.8	28.6	20.8
Cancer	9.1	10.1	7.9	8.5	9.8	7.1
CHF	2.3	2.2	2.5	2.1	1.8	2.4
Chronic liver disease	3.2	2.8	3.7	3.3	2.7	3.9
Chronic lung disease	7.1	8.8	5.3	7.2	8.9	5.5
CAD	3.3	2.3	4.5	3.3	1.9	4.8
Diabetes	8.3	8.3	8.3	7.4	7.2	7.6
Stroke	2.6	3.0	2.3	2.6	3.0	2.1
Thyroid problem	9.6	15.2	3.6	9.4	14.7	3.8

% and median [interquartile range] are reported. There were missing values in the BMI cohort: HOMA-IR (58.1%), fasting insulin (58.0%), fasting glucose (57.3%), CRP (30.5%), HbA1c (4.8%), smoking status (<0.1%), number of chronic conditions (<0.1%), and other individual morbidities (<0.1%). There were missing values in the cohort: HbA1c (0.2%), fasting glucose (0.2%), HOMA-IR (0.2%), smoking status (<0.1%), number of chronic conditions (<0.1%), and diabetes (<0.1%).

*BMI* body mass index, *CAD* coronary artery disease, *CHF* chronic heart failure, *CRP* c-reactive protein, *HbA1c* glycated hemoglobin, *HOMA-IR* Homeostatic Model Assessment of Insulin Resistance.

Over median follow-up of 96 months [IQR 52,144] and 123 months [IQR 88,161] for the BMI and primary cohorts, there were 3616 (8.9%) and 1342 (10.7%) deaths, respectively.

### BMI and all-cause mortality in the BMI cohort

**Fig. 1:**
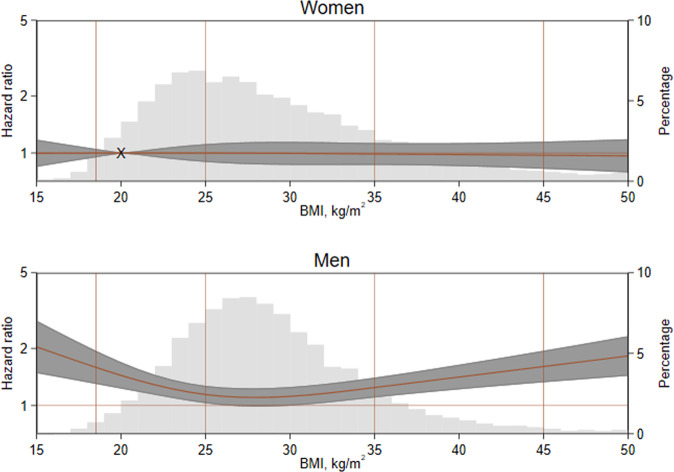
Hazard ratios of mortality by body mass index and biological sex. BMI body mass index The rightmost *y*-axis shows the percentages for the distribution of body mass index. The hazard ratios for mortality are shown on the leftmost *y*-axis. The referent (hazard ratio of 1) is a woman in with a BMI of 20 kg/m^2^; it is marked with an X and a horizontal line is drawn through it. The two left-most vertical lines indicate the theoretical range of the so-called “healthy” BMIs: 18.5–25 kg/m^2^. In this BMI cohort, 3616 (8.9%) of participants died.

In sex-adjusted models, the association between BMI (parametrized with RCS) and mortality was concave U-shaped in men and flatly linear in women (Fig. 1) where the minima for men was 28 kg/m^2^, corresponding to overweight in those without Asian ancestry [25]. The distribution of participants in these sex groups was right-tailed with larger tails evident in the female population (standard error was 0.079 in women vs 0.068 kg/m^2^ in men).

### BMI, fasting insulin, and CRP in the primary cohort

**Fig. 2:**
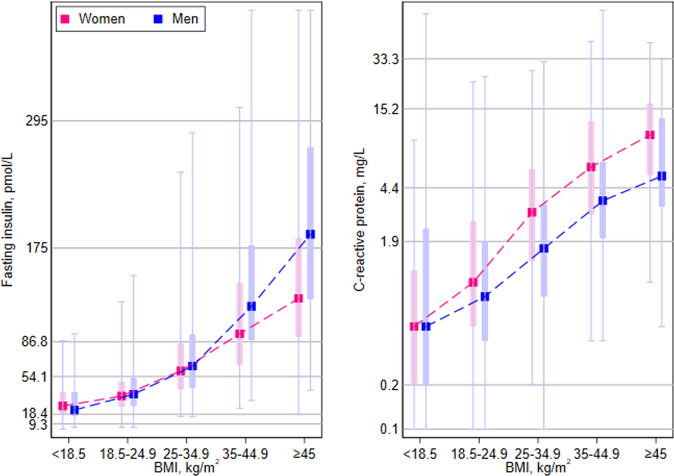
Fasting insulin and c-reactive protein by categories of body mass index and biological sex. BMI body mass index The square markers show the median fasting insulin (left panel) and c-reactive protein (right panel) levels by biological sex (women in pink, men in blue) by body mass index. The vertical bars show the interquartile ranges, and the vertical lines show the range from the 1st percentile to the 99th percentile by body mass index. The gray horizontal lines in the left panel show the 1st, 5th, 50th, 75th, 95th, and 99th percentiles for fasting insulin in this study population. The gray horizontal lines in the right panel show the 1st, 5th, 50th, 75th, 95th, and 99th percentiles for c-reactive protein in this study population. These are data from the primary cohort. The curves of fasting insulin between the sexes start to separate at BMIs between 18.5 and 24.9 kg/m^2^. The *p* values using gamma regression were 0.96 for <18.5, 0.009 for 18.5–24.9, <0.001 for 25-34.9, <0.001 for 35–44.9, and 0.002 for ≥45 kg/m^2^. The curves of c-reactive protein between the sexes start to separate at BMIs between 18.5 and 25.9 kg/m^2^. The *p* values using Gaussian regression were 0.64 for <18.5, <0.001 for 18.5–24.9, <0.001 for 25–34.9, <0.001 for 35–44.9, and 0.004 for ≥45 kg/m^2^.

Both fasting insulin and CRP correlated with BMI (0.40 and 0.21 [or 0.42 if using the natural logarithm of CRP]; Fig. 2). While the proportion of those in the top 25th percentile of fasting insulin (≥87 pmol//L) increased with increasing BMI (from 1.3% to 81.3%), there were participants in all five BMI categories with fasting insulin level in the top 25th percentile (Supplement Fig. S2). While the proportion of those in the top 25th percentile of CRP (≥4.4 mg/L) increased with increasing BMI (from 6.0% to 74.5%), there were participants in all five BMI categories with CRP levels in the top 25th percentile.

The curves in Fig. 2 separated by sex with men having higher fasting insulin levels at higher BMI (beginning at 18.5–24.9 kg/m^2^, *p* = 0.009), and women having higher CRP levels at higher BMI (beginning at 18.5–24.9 kg/m^2^, *p* < 0.001).

### BMI, fasting insulin, CRP, and all-cause mortality in the primary cohort

**Fig. 3:**
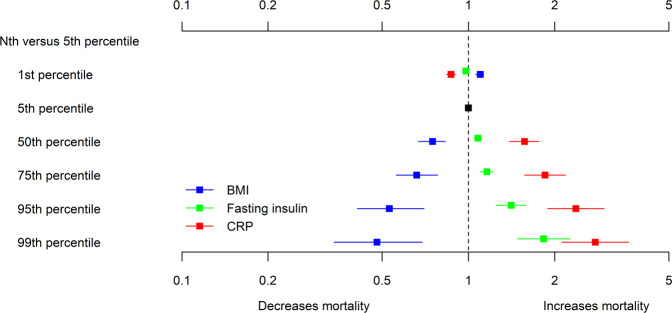
Adjusted hazard ratios of mortality by percentiles of body mass index, fasting insulin, and c-reactive protein. BMI body mass index, CRP c-reactive protein These hazards ratios are adjusted for body mass index (a quadratic and linear term), fasting insulin (a linear term), c-reactive protein (natural logarithm term), age, biological sex, smoking status, and ten chronic conditions (angina, arthritis, cancer, chronic heart failure, chronic liver disease, chronic lung disease, coronary artery disease, diabetes, stroke, and thyroid problems). The black marker represents the referent at the 5th percentile for all three exposures of interest. In this primary cohort, 1342 (10.7%) of participants died.

After adjustment for age, sex, and smoking (Model 1), there was no evidence of an association between BMI and mortality (Table 2). After additional adjustment for fasting insulin and CRP (Model 2), higher BMI was associated with lower mortality (from an HR of 1.08, 95% CI 1.04,1.12 at the 1st percentile to an HR of 0.56, 95% CI 0.40,0.78 at the 99th percentile, both compared to the 5th percentile). In this model, higher fasting insulin and CRP were each associated with higher risks for mortality (fasting insulin: from an HR of 0.98, 95% CI 0.97,0.98 for the 1st percentile to an HR of 2.07, 95% CI 1.71,2.51 for the 99th percentile; and CRP from an HR of 0.86, 95% CI 0.83,0.90 for the 1st percentile to an HR of 2.93, 95% CI 2.21, 3.88 for the 99th percentile). These associations remained present after further adjustment for the 10 chronic conditions (Model 3; Fig. 3). The associations with mortality mildly strengthened for BMI, and mildly attenuated for fasting insulin and CRP.

**Table 2:** Adjusted hazard ratios of mortality by percentiles of body mass index, fasting insulin, and c-reactive protein.

Characteristic	Hazard ratio (95% confidence interval)		
	Model 1 – Age, sex, smoking history, BMI	Model 2 – Plus fasting insulin, CRP	Model 3 – Plus 10 chronic conditions
Body mass index, kg/m^2^			
18 vs 20	1.01 (0.98,1.04)	1.08 (1.04,1.12)	1.10 (1.06,1.13)
27 vs 20	0.99 (0.90,1.08)	0.79 (0.71,0.88)	0.75 (0.67,0.83)
32 vs 20	0.99 (0.86,1.15)	0.71 (0.60,0.83)	0.66 (0.56,0.78)
41 vs 20	1.06 (0.84,1.33)	0.60 (0.46,0.78)	0.53 (0.41,0.70)
48 vs 20	1.17 (0.87,1.57)	0.56 (0.40,0.78)	0.48 (0.34,0.69)
Fasting insulin, pmol/L	-		
9 vs 18		0.98 (0.97,0.98)	0.98 (0.97,0.99)
54 vs 18		1.10 (1.07,1.13)	1.08 (1.05,1.11)
87 vs 18		1.20 (1.14,1.26)	1.16 (1.10,1.22)
175 vs 18		1.51 (1.36,1.69)	1.41 (1.25,1.59)
295 vs 18		2.07 (1.71,2.51)	1.83 (1.48,2.26)
C-reactive protein, mg/L	**-**		
0.1 vs 0.2		0.86 (0.83,0.90)	0.87 (0.84,0.90)
1.9 vs 0.2		1.60 (1.42,1.82)	1.57 (1.39,1.76)
4.4 vs 0.2		1.91 (1.61,2.27)	1.85 (1.57,2.18)
15.2 vs 0.2		2.48 (1.96,3.15)	2.37 (1.89,2.97)
33.3 vs 0.2		2.93 (2.21,3.88)	2.77 (2.12,3.62)

Hazard ratios with 95% confidence intervals are presented for the 1st, 50th, 75th, 95th, and 99th vs 5th percentile for body mass index, fasting insulin, and c-reactive protein. Model 1 adjusts for body mass index (a quadratic and linear term), age, biological sex, and smoking status. Model 2 adjusts for body mass index (a quadratic and linear term), fasting insulin (a linear term), c-reactive protein (a natural logarithm term), age, biological sex, and smoking status. Model 3 adjusts for body mass index (a quadratic and linear term), fasting insulin (a linear term), c-reactive protein (natural logarithm term), age, biological sex, smoking status, and 10 chronic conditions (angina, arthritis, cancer, chronic heart failure, chronic liver disease, chronic lung disease, coronary artery disease, diabetes, stroke, and thyroid problems). In this primary cohort, 1,342 (10.7%) participants died.

Results were similar in sensitivity analyses with further adjustment for HbA1c and separately for fasting glucose (Supplement Table S1). Alternative sensitivity analyses that adjusted for the HOMA-IR rather than fasting insulin, and for percentage fat mass rather than BMI, also did not importantly change the results.

When we considered every combination of adjusting and not adjusting for the 3 main exposures (BMI, fasting insulin, CRP; Supplement Table S2), the inverse associations of BMI with mortality were consistently stronger when fasting insulin and/or CRP were included in the model.

In subgroup analyses (Supplement Table S3), the grade of inflammation modified the association between BMI and mortality (*P* for interaction = 0.0002). The inverse association between BMI and mortality was stronger for participants with versus without a moderate or high grade of inflammation. Additionally, the inverse association between BMI and mortality appeared stronger for women than for men (*P* for interaction = 0.046).

## Discussion

After accounting for levels of fasting insulin and CRP, we found no evidence of an association between higher BMI and increased mortality. Instead, higher fasting insulin and higher CRP were each associated with an excess risk of mortality. In models that simultaneously accounted for BMI, fasting insulin, and CRP, participants with higher BMI had lower risks of mortality, and participants with higher fasting insulin and/or CRP levels had higher risks of mortality, indicating that fasting insulin and CRP confound the association between BMI and mortality. Additionally, among people with higher BMI, CRP tended to be higher among women than in men, whereas the converse was true for fasting insulin.

Prior work showing that obesity is associated with excess mortality generally has not accounted for the potential role of hyperinsulinemia or chronic inflammation. Shi et al. [26] used the same dataset as in the current study to explore the potential confounding effects of metabolic syndrome (defined by higher levels of waist circumference, triglycerides, HDL-cholesterol, blood pressure, and fasting glucose) on the association between BMI and mortality. Although they did not consider fasting insulin or CRP, after adjustment for these other potential confounders, Shi et al. also found an inverse association between BMI and mortality that was strongest for those with metabolic syndrome, which is broadly consistent with our findings.

Collider bias has been cited as a potential explanation for analyses showing that obesity is associated with lower mortality in people with chronic diseases. This form of bias arises when the exposure or the outcome is used to select participants for inclusion in analyses, and so would not have affected our findings, which are based on a representative sample of the general US population.

We found that the lower risk of mortality associated with higher BMI was consistent in models that included fasting insulin or CRP (Supplement Table S2). This finding is not compatible with the common view that these characteristics mediate an association between BMI and excess mortality. Rather, our findings suggest that fasting insulin and CRP confound the association between BMI and mortality—and that after accounting for the higher risk of mortality that would be expected to accompany higher levels of insulin or CRP, higher BMI was associated with lower mortality.

This hypothesis is plausible, because available evidence in humans suggests that hyperinsulinemia precedes weight gain (rather than the other way around) [20], and that adiposity amplifies inflammation but that adiposity does not initiate the inflammatory cascade [27]. In our study, 13.4% of participants with BMI ≥ 30 kg/m^2^ had CRP ≤ 1 mg/L, which further highlights that inflammation initiation is not necessarily a consequence or a correlate of obesity. While most people with hyperinsulinemia and chronic inflammation do have higher BMI, our data show that a subset of people with lower BMI also have hyperinsulinemia and/or chronic inflammation (Fig. 2), and that these participants are at the highest risk of mortality.

Current public health strategies have tended to highlight the importance of weight reduction. At the population level, these strategies have increased weight stigma [28–30], weight cycling [31] and weight gain [32, 33]—all of which associate with increased inflammation and hyperinsulinemia. Our findings suggest the need for renewed focus on alternative interventions that reduce levels of insulin [34, 35], inflammation [36, 37], and perhaps the link between physiological stress, hyperinsulinemia, and inflammation [38–42].

### Limitations

This study had a number of strengths. While the dataset was small, there was sufficient power to detect associations between the main exposures and mortality in a sample that is representative of the general adult US population. There are also a number of limitations within this dataset. First, there was only one measure of CRP, which could have led to misclassification of some participants with a self-limited condition such as a viral infection. However, this misclassification would be expected to bias toward the null and so availability of a second measure of CRP is unlikely to have influenced our conclusions. Second, there was no data on weight cycling [31] or weight-based discrimination [30] for people with high BMI, which might have influenced the association between BMI and mortality [30]. Third, this study could not explore the temporal associations between BMI, hyperinsulinemia, chronic inflammation, and the development of noncommunicable chronic disease such as hypertension, cancer, diabetes, or coronary disease, as mortality is the only longitudinal outcome available in this dataset.

In conclusion, higher fasting insulin and higher CRP each confound the association between BMI and the risk of all-cause mortality. Therefore, the increase in mortality that has been attributed to obesity is more likely due to hyperinsulinemia and inflammation.

## Disclaimer

This study’s data are provided by the National Centre for Health Statistics. The interpretation and conclusions contained herein are those of the researchers and do not represent the views of the NCHS. The NCHS expresses any opinion in relation to this study.

## Supplementary information

Supplement

## Data Availability

These data are publicly available from https://www.cdc.gov/nchs/nhanes/index.htm.
