# Supplementary material for: Associations of body mass index, fasting insulin, and inflammation with mortality: a prospective cohort study
Source: Int J Obes (Lond). 2022 Aug 27;46(12):2107–13. doi: 10.1038/s41366-022-01211-2 (PMC9678791; doi:10.1038/s41366-022-01211-2)
Supplement: Supplementary file 1 — Supplement [file 41366_2022_1211_MOESM1_ESM.docx]

**Figure S1. Sample participant flow**

**
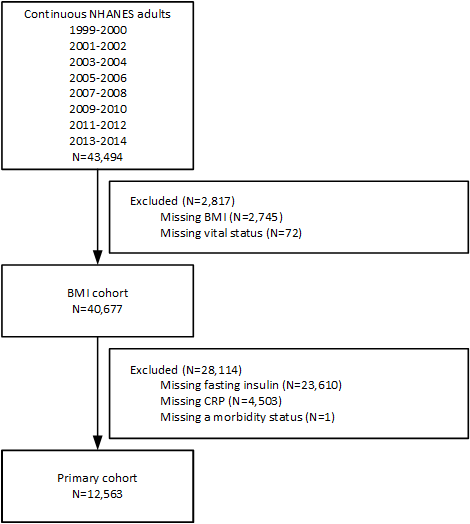
**

BMI body mass index, CRP c-reactive protein, NHANES National Health and Nutrition Examination Survey

**Supplement Figure S2. Distribution of body mass index in participants with high fasting insulin and high c-reactive protein**

The gray shade of the pie slice indicates the category of body mass index: <18.5 (darkest), 18.5-24.9, 25-34.9, 35-44.9, ≥45 kg/m^2^ (lightest). The slices are labelled with the percentages of participants in the ≥75^th^ percentile of fasting insulin (in the left panel), or c-reactive protein (in the right panel). These are data from the primary cohort.

**Supplement Table S1. Adjusted hazard ratios of mortality by percentiles of body mass index, fasting insulin and c-reactive protein – sensitivity analyses**

| **Characteristic** | **Hazard ratio (95% confidence interval)** | | | | |
| --- | --- | --- | --- | --- | --- |
|  | **Model 3** | **Model 3 –**  **Add A1c** | **Model 3 –**  **Add fasting glucose** | **Model 3 –**  **Replace fasting**  **insulin with**  **HOMA-IR** | **Model 3 –**  **Replace BMI with**  **% fat mass** |
| Body mass index, kg/m^2^ |  |  |  |  |  |
| 18 vs 20 | 1.10 (1.06,1.13) | 1.10 (1.06,1.14) | 1.10 (1.06,1.14) | 1.09 (1.06,1.13) | 1.11 (0.99,1.26)^2^ |
| 27 vs 20 | 0.75 (0.67,0.83) | 0.74 (0.67,0.83) | 0.74 (0.66,0.83) | 0.76 (0.68,0.85) | 0.69 (0.50,0.96) |
| 32 vs 20 | 0.66 (0.56,0.78) | 0.65 (0.55,0.77) | 0.64 (0.54,0.76) | 0.67 (0.57,0.79) | 0.60 (0.40,0.90) |
| 41 vs 20 | 0.53 (0.41,0.70) | 0.52 (0.40,0.68) | 0.52 (0.39,0.68) | 0.55 (0.42,0.72) | 0.51 (0.33,0.80) |
| 48 vs 20 | 0.48 (0.34,0.69) | 0.47 (0.33,0.67) | 0.46 (0.32,0.66) | 0.50 (0.36,0.72) | 0.46 (0.29,0.75) |
| Fasting insulin, pmol/L |  |  |  |  |  |
| 9 vs 18 | 0.98 (0.97,0.99) | 0.98 (0.97,0.99) | 0.98 (0.97,0.99) | 0.99 (0.98,0.99)^1^ | 0.98 (0.97,0.99) |
| 54 vs 18 | 1.08 (1.05,1.11) | 1.08 (1.05,1.11) | 1.08 (1.05,1.11) | 1.06 (1.04,1.07) | 1.08 (1.05,1.11) |
| 87 vs 18 | 1.16 (1.10,1.22) | 1.16 (1.10,1.22) | 1.16 (1.10,1.22) | 1.12 (1.08,1.15) | 1.16 (1.09,1.22) |
| 175 vs 18 | 1.41 (1.25,1.59) | 1.40 (1.24,1.58) | 1.39 (1.24,1.57) | 1.32 (1.22,1.43) | 1.40 (1.22,1.59) |
| 295 vs 18 | 1.83 (1.48,2.26) | 1.81 (1.46,2.23) | 1.79 (1.45,2.22) | 1.77 (1.51,2.07) | 1.80 (1.43,2.27) |
| C-reactive protein, mg/L |  |  |  |  |  |
| 0.1 vs 0.2 | 0.87 (0.84,0.90) | 0.87 (0.84,0.91) | 0.87 (0.84,0.91) | 0.87 (0.84,0.90) | 0.88 (0.84,0.91) |
| 1.9 vs 0.2 | 1.57 (1.39,1.76) | 1.55 (1.38,1.75) | 1.55 (1.38,1.75) | 1.57 (1.39,1.76) | 1.53 (1.35,1.73) |
| 4.4 vs 0.2 | 1.85 (1.57,2.18) | 1.83 (1.56,2.15) | 1.83 (1.56,2.15) | 1.85 (1.57,2.17) | 1.80 (1.52,2.13) |
| 15.2 vs 0.2 | 2.37 (1.89,2.97) | 2.33 (1.86,2.92) | 2.33 (1.86,2.93) | 2.37 (1.89,2.97) | 2.27 (1.79,2.89) |
| 33.3 vs 0.2 | 2.77 (2.12,3.62) | 2.72 (2.08,3.55) | 2.72 (2.08,3.56) | 2.77 (2.12,3.61) | 2.64 (1.99,3.50) |

A1c glycated hemoglobin, BMI body mass index, HOMA-IR Homeostatic Model Assessment of Insulin Resistance

^1^These are percentile comparisons of the HOMA-IR and not fasting insulin.

^2^These are percentile comparisons of the percentage of fat mass and not body mass index.

Hazard ratios with 95% confidence intervals are presented for the 1^st^, 50^th^, 75^th^, 95^th^, and 99^th^ vs 5^th^ percentile for body mass index, fasting insulin (or HOMA-IR) and c-reactive protein. Model 3 adjusts for body mass index (a quadratic and linear term), fasting insulin (a linear term), c-reactive protein (natural logarithm term), age, biological sex, smoking status and 10 chronic conditions (angina, arthritis, cancer, chronic heart failure, chronic liver disease, chronic lung disease, coronary artery disease, diabetes, stroke and thyroid problems). There are 4 sensitivity analyses: 1) adding A1c (linear term) to Model 3, 2) adding fasting glucose (linear term) to Model 3, 3) replacing fasting insulin with HOMA-IR (linear term), and 4) replacing body mass index with percentage of fat mass. In this primary cohort, 10.7% of participants died.

**Supplement Table S2. Adjusted hazard ratios of mortality by percentiles of body mass index, fasting insulin and c-reactive protein – permutation analyses**

| **Characteristic** | **Hazard ratio (95% confidence interval)** | | | | | | |
| --- | --- | --- | --- | --- | --- | --- | --- |
|  | **Model 3** | **Model 3 –**  **No fasting insulin, no CRP** | **Model 3 –**  **No BMI, no CRP** | **Model 3 –**  **No BMI, no fasting insulin** | **Model 3 –**  **No fasting insulin** | **Model 3 –**  **No CRP** | **Model 3 –**  **No BMI** |
| Body mass index, kg/m^2^ |  |  | - | - |  |  | - |
| 18 vs 20 | 1.10 (1.06,1.13) | 1.04 (1.01,1.07) |  |  | 1.07 (1.03,1.10) | 1.07 (1.03,1.10) |  |
| 27 vs 20 | 0.75 (0.67,0.83) | 0.91 (0.82,1.0002) |  |  | 0.82 (0.74,0.91) | 0.83 (0.74,0.92) |  |
| 32 vs 20 | 0.66 (0.56,0.78) | 0.87 (0.75,1.02) |  |  | 0.75 (0.64,0.87) | 0.76 (0.65,0.90) |  |
| 41 vs 20 | 0.53 (0.41,0.70) | 0.86 (0.67,1.10) |  |  | 0.66 (0.51,0.85) | 0.69 (0.52,0.90) |  |
| 48 vs 20 | 0.48 (0.34,0.69) | 0.90 (0.65,1.24) |  |  | 0.64 (0.46,0.88) | 0.67 (0.47,0.96) |  |
| Fasting insulin, pmol/L |  | - |  | - | **-** |  |  |
| 9 vs 18 | 0.98 (0.97,0.99) |  | 0.98 (0.98,0.99) |  |  | 0.98 (0.97,0.99) | 0.99 (0.98,0.99) |
| 54 vs 18 | 1.08 (1.05,1.11) |  | 1.07 (1.04,1.10) |  |  | 1.08 (1.05,1.11) | 1.05 (1.02,1.09) |
| 87 vs 18 | 1.16 (1.10,1.22) |  | 1.13 (1.08,1.19) |  |  | 1.16 (1.11,1.23) | 1.11 (1.04,1.18) |
| 175 vs 18 | 1.41 (1.25,1.59) |  | 1.34 (1.19,1.50) |  |  | 1.42 (1.26,1.59) | 1.26 (1.10,1.45) |
| 295 vs 18 | 1.83 (1.48,2.26) |  | 1.67 (1.35,2.05) |  |  | 1.85 (1.51,2.28) | 1.51 (1.18,1.93) |
| C-reactive protein, mg/L |  | **-** | **-** |  |  | **-** |  |
| 0.1 vs 0.2 | 0.87 (0.84,0.90) |  |  | 0.88 (0.85,0.91) | 0.87 (0.84,0.90) |  | 0.89 (0.86,0.93) |
| 1.9 vs 0.2 | 1.57 (1.39,1.76) |  |  | 1.50 (1.33,1.68) | 1.58 (1.41,1.78) |  | 1.45 (1.29,1.64) |
| 4.4 vs 0.2 | 1.85 (1.57,2.18) |  |  | 1.74 (1.49,2.04) | 1.88 (1.60,2.20) |  | 1.67 (1.41,1.97) |
| 15.2 vs 0.2 | 2.37 (1.89,2.97) |  |  | 2.17 (1.74,2.71) | 2.42 (1.93,3.03) |  | 2.05 (1.62,2.58) |
| 33.3 vs 0.2 | 2.77 (2.12,3.62) |  |  | 2.50 (1.93,3.25) | 2.84 (2.17,3.70) |  | 2.33 (1.77,3.06) |

BMI body mass index, CRP c-reactive protein

Hazard ratios with 95% confidence intervals are presented for the 1^st^, 50^th^, 75^th^, 95^th^, and 99^th^ vs 5^th^ percentile for body mass index, fasting insulin and c-reactive protein. Model 3 adjusts for body mass index (a quadratic and linear term), fasting insulin (a linear term), c-reactive protein (natural logarithm term), age, biological sex, smoking status and 10 chronic conditions (angina, arthritis, cancer, chronic heart failure, chronic liver disease, chronic lung disease, coronary artery disease, diabetes, stroke and thyroid problems). The following models adjust for every permutation of body mass index (included/excluded), fasting insulin (included/excluded), and c-reactive protein (included/excluded). This sample represented 201,109,631 people where the estimated percentage of deaths was 10.7%. In this primary cohort, 10.7% of participants died.

**Supplement Table S3. Adjusted hazard ratios of mortality by percentiles of body mass index, fasting insulin and c-reactive protein – subgroup analyses**

| **Characteristic** | **Hazard ratio (95% confidence interval)** | | | | |
| --- | --- | --- | --- | --- | --- |
|  | **Model 3** | **Model 4** |  | **Model 5** |  |
|  |  | **CRP ≤10 mg/L** | **CRP >10 mg/L** | **Women** | **Men** |
| Deaths, % | 10.7 | 10.1 | 16.3 | 9.4 | 12.0 |
| Body mass index, kg/m^2^ |  |  |  |  |  |
| 18 vs 20 | 1.10 (1.06,1.13) | 1.11 (1.07,1.16) | 1.12 (1.06,1.18) | 1.16 (1.10,1.23) | 1.08 (1.02,1.14) |
| 27 vs 20 | 0.75 (0.67,0.83) | 0.73 (0.66,0.82) | 0.71 (0.59,0.85) | 0.65 (0.55,0.76) | 0.80 (0.66,0.96) |
| 32 vs 20 | 0.66 (0.56,0.78) | 0.64 (0.55,0.76) | 0.60 (0.46,0.79) | 0.55 (0.44,0.68) | 0.72 (0.54,0.95) |
| 41 vs 20 | 0.53 (0.41,0.70) | 0.56 (0.42,0.75) | 0.46 (0.30,0.71) | 0.46 (0.44,0.68) | 0.61 (0.38,0.98) |
| 48 vs 20 | 0.48 (0.34,0.69) | 0.58 (0.35,0.94) | 0.40 (0.23,0.70) | 0.49 (0.32,0.74) | 0.57 (0.31,0.98) |
| Fasting insulin, pmol/L |  |  |  |  |  |
| 9 vs 18 | 0.98 (0.97,0.99) | 0.98 (0.97,0.99) | 0.98 (0.96,0.998) | 0.98 (0.97,0.99) | 0.98 (0.97,0.99) |
| 54 vs 18 | 1.08 (1.05,1.11) | 1.08 (1.05,1.11) | 1.08 (1.01,1.17) | 1.10 (1.06,1.15) | 1.07 (1.04,1.11) |
| 87 vs 18 | 1.16 (1.10,1.22) | 1.16 (1.10,1.22) | 1.17 (1.02,1.34) | 1.20 (1.11,1.31) | 1.14 (1.07,1.22) |
| 175 vs 18 | 1.41 (1.25,1.59) | 1.40 (1.24,1.59) | 1.43 (1.04,1.96) | 1.53 (1.27,1.84) | 1.36 (1.18,1.56) |
| 295 vs 18 | 1.83 (1.48,2.26) | 1.82 (1.46,2.26) | 1.87 (1.07,3.28) | 2.12 (1.53,2.94) | 1.71 (1.33,2.20) |
| C-reactive protein, mg/L |  |  |  |  |  |
| 0.1 vs 0.2 | 0.87 (0.84,0.90) | 0.88 (0.84,0.93) | - | 0.88 (0.83,0.93) | 0.87 (0.82,0.91) |
| 1.9 vs 0.2 | 1.57 (1.39,1.76) | 1.50 (1.27,1.77) | - | 1.53 (1.26,1.86) | 1.60 (1.36,1.88) |
| 4.4 vs 0.2 | 1.85 (1.57,2.18) | 1.75 (1.39,2.20) | - | 1.79 (1.37,2.34) | 1.91 (1.53,2.38) |
| 15.2 vs 0.2 | 2.37 (1.89,2.97) | - | 2.19 (1.59,3.01) | 2.26 (1.55,3.29) | 2.47 (1.82,3.36) |
| 33.3 vs 0.2 | 2.77 (2.12,3.62) | - | 2.52 (1.73,3.68) | 2.62 (1.68,4.08) | 2.91 (2.02,4.19) |

Hazard ratios with 95% confidence intervals are presented for the 1^st^, 50^th^, 75^th^, 95^th^, and 99^th^ vs 5^th^ percentile for body mass index, fasting insulin and c-reactive protein. Model 4 adjusts for all covariates in model 3 plus interactions terms between CRP (categorized as ≤10 mg/L vs >10 mg/L) and body mass index, fasting insulin and c-reactive protein. CRP grade did significantly modify the association between body mass index and mortality but CRP grade did not significantly modify the association between fasting insulin and mortality. Model 5 adjusts for all covariates in model 3 plus interactions terms between biological sex and body mass index, fasting insulin and c-reactive protein. Biological sex did significantly modify the association between body mass index and mortality but sex did not significantly modify the association between both fasting insulin and CRP, with mortality. Model 6 adjusts for all covariates in model 3 plus interactions terms between age categories (<50, 50-69, ≥70 years) and body mass index, fasting insulin and c-reactive protein. Age did significantly modify the association between both body mass index and fasting insulin, with mortality but not CRP. These are data from the primary cohort.
